# Nonsteroidal Anti-Inflammatory Drug-Induced Granulomatous Colitis: A Case Report and Literature Review of a Vanishing Colonic Mass Mimicking Malignancy

**DOI:** 10.1155/crgm/1169139

**Published:** 2025-08-20

**Authors:** Joel Gabin Konlack Mekontso, Akil Olliverrie, Nitin Pendyala, Joseph Yvan Bena Nnang, Guy Loic Nguefang Tchoukeu, Vera Platsky, Roy Chaudhury, Christopher Chum

**Affiliations:** ^1^Department of Internal Medicine, New York City Health and Hospitals South Brooklyn Health, Brooklyn, New York, USA; ^2^Department of General Medicine, Faculty of Medicine and Biomedical Sciences, University of Yaounde I, Yaounde, Cameroon; ^3^Department of Internal Medicine, Texas Tech University Health Sciences Center School of Medicine, Odessa, Texas, USA; ^4^Department of Pathology, New York City Health and Hospitals South Brooklyn Health, Brooklyn, New York, USA; ^5^Department of Gastroenterology, New York City Health and Hospitals South Brooklyn Health, Brooklyn, New York, USA

**Keywords:** colon carcinoma, colon mass, granulomatous mass, ischemic colitis, nonsteroidal anti-inflammatory drugs

## Abstract

This case report describes a rare occurrence of nonsteroidal anti-inflammatory drugs (NSAID)–induced focal colonic granulomatous mass mimicking a malignant colonic mass. It highlights the diagnostic challenges of NSAID-related gastrointestinal complications and stresses the importance of considering such causes in similar presentations. Prompt recognition and withdrawal of the offending NSAID can prevent unnecessary surgical intervention and facilitate symptom resolution. This case underscores the value of a detailed medication history and cautious NSAID use to reduce the risk of adverse gastrointestinal effects.

## 1. Introduction

Nonsteroidal anti-inflammatory drugs (NSAIDs) are among the most commonly prescribed medications globally, known for their effectiveness in managing pain and inflammation. While their association with upper gastrointestinal (GI) complications such as gastritis, ulcers, and bleeding are well documented, lower GI complications have gained attention more recently due to advancements in diagnostic techniques [1]. Despite this increased awareness, NSAID-induced colonic granulomatous masses remain a rare and under-reported entity.

## 2. Case Presentation

A previously healthy 63-year-old woman presented with a four-day history of nonbloody, nonbilious vomiting and diarrhea. She denied constitutional symptoms, rash, or recent dietary changes. Her medical history included well-controlled hypertension, hyperlipidemia, diabetes, and hepatitis C treated 14 years prior with recent evidence of sustained virologic response. She was taking losartan 100 mg daily, nifedipine ER 90 mg daily, atorvastatin 20 mg daily, and metformin 1000 mg twice daily. Notably, she had started over-the-counter ibuprofen “several weeks ago” to manage arthritis pain. She denied recent use of antibiotics, herbal remedies, or any known allergies and had a 10-pack-year smoking history. A colonoscopy 3 years prior was normal.

**Figure 1:**
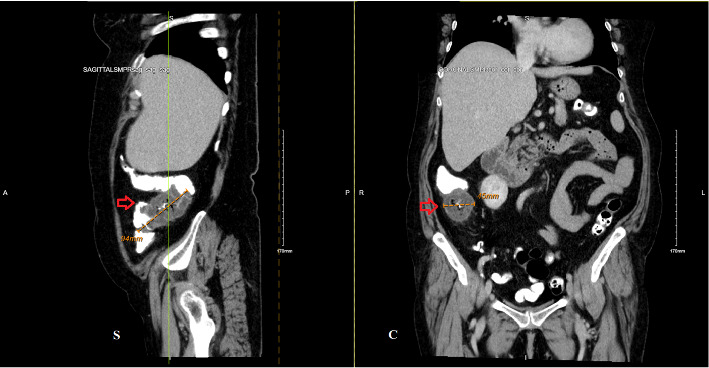
Contrast-enhanced abdominal computed tomography demonstrates an annular mass within the proximal ascending colon (red arrows in sagittal (S) and coronal (C) sections), measuring 94 mm in length and 45 mm in diameter with overhanging edges and subtle stranding of the pericolonic fat. Multiple subcentimeter adjacent lymph nodes are noted, not depicted on the provided images.

**Figure 2:**
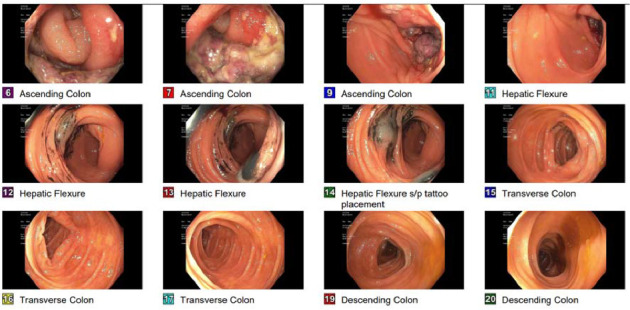
Colonoscopic view showing a polypoid, ulcerated mass in the ascending colon.

**Figure 3:**
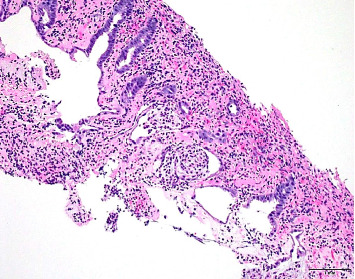
Biopsy of the ascending colon mucosal mass revealing inflammatory and ischemic changes (small glands, edematous, and fibrinous lamina propria with pseudomembranes' formation). Hematoxylin and eosin stain, ×200 magnification.

**Figure 4:**
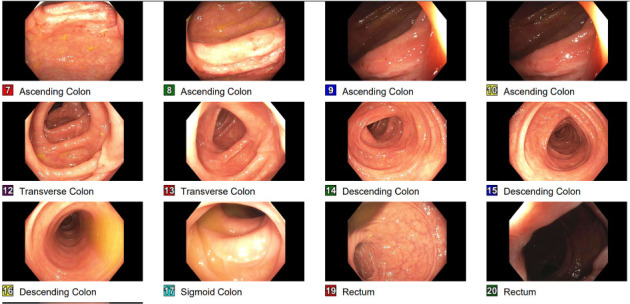
Repeat colonoscopy 6 days later showing normal ascending colon.

**Figure 5:**
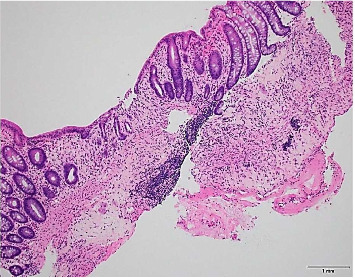
Repeat biopsy of the ascending colonic mucosa demonstrating interval improvement in inflammatory infiltrate with crypt dropout, focal crypt architectural distortion, and lamina propria hyalinization (hematoxylin and eosin stain, ×200 magnification).

Physical examination and initial laboratory tests were unremarkable. The patient was appropriately resuscitated, and ibuprofen was discontinued upon admission. Abdominal computed tomography (CT) revealed a suspicious mass measuring approximately 94 × 45 mm in the proximal ascending colon, raising concerns for colon adenocarcinoma (Figure 1). Gastroenterology consultation prompted a colonoscopy, which identified an infiltrative, polypoid, ulcerated mass in the ascending colon (Figure 2). Biopsy results demonstrated acute and chronic colitis, possibly due to infectious or drug-induced ischemic etiologies (Figure 3). These findings conflicting with the strong clinical suspicion of malignancy raised concerns about inadequate specimen collection, prompting a second colonoscopy 6 days later, which ultimately revealed complete endoscopic resolution of the mass (Figure 4) and confirmed its benign nature, with only inflammatory changes on histology (Figure 5). The patient was discharged symptom free, resumed her chronic medications, and was strongly advised to avoid NSAIDs. At a 3-month follow-up, she remained asymptomatic. A repeat abdominopelvic CT scan performed the day before the appointment showed no evidence of a colonic mass. A subsequent colonoscopy revealed a healed colonic ulceration at the previously tattooed site but was, otherwise, unremarkable.

## 3. Discussion

NSAIDs, widely used medications, are categorized as selective or nonselective based on their cyclooxygenase (COX) enzyme inhibition. While selective NSAIDs primarily inhibit COX-2, nonselective NSAIDs inhibit both COX-1 and COX-2, leading to GI side effects due to COX-1's role in protecting the GI mucosa [1, 2]. Although serious GI complications occur in only 1%-2% of NSAID users, advancements in diagnostic techniques have revealed an increasing prevalence of lower GI complications, now accounting for at least 40% of all NSAID-related serious GI events [1, 2]. Age over 65 is a significant risk factor. The toxicity of nonselective NSAIDs varies, with some being more damaging to the GI mucosa than others, while selective NSAIDs generally appear safer. However, COX-1 inhibition is not the only mechanism contributing to GI toxicity. Increasing evidence is available about mechanisms of action independent of COX inhibition. These factors include direct disruption of phospholipid layers leading to increased permeability, endoplasmic reticulum and mitochondrial dysfunction with the impairment of oxidative phosphorylation leading to free-radical production, and disruption of the gut microbiota [2].

Lower GI complications of NSAIDs include mucosal ulceration, bleeding, inflammation, anemia, malabsorption, and rarely, ischemia-induced granuloma formation, as seen in our case. They have also been associated with relapse of inflammatory bowel disease, eosinophilic allergic colitis, collagenous colitis, segmental ischemia, and diaphragm-like strictures [1, 3]. While NSAIDs are a well-established cause of drug-induced colitis, with a prevalence of 16.6%, other drug classes, such as angiotensin II receptor blockers (9.5%), metformin (3.8%), and statins (4%), have also been linked to this condition [4]. Although our patient's presentation could be related to her other medications, we strongly suspect ibuprofen as the trigger as her symptoms arose following its recent introduction. Importantly, restarting metformin, losartan, and atorvastatin upon discharge did not elicit any serious adverse reactions. Diabetes could theoretically heighten gut vulnerability to inflammation through altered motility, impaired visceral sensation, microischemia, and poor wound healing. However, this was likely not a significant factor for our patient, given her consistently well-controlled diabetes (HbA1c < 6.5%). Furthermore, our literature search found no comparative studies on the prevalence or severity of NSAID-induced colitis in diabetic versus nondiabetic populations.

NSAID-induced mass-forming granulomatous colitis can mimic malignant colonic lesions, presenting a significant diagnostic challenge. Granulomas form due to immune responses involving macrophages and multinucleated giant cells. NSAIDs increase mucosal permeability and inflammation in up to 60%–70% of the users, allowing antigens to induce a type IV hypersensitivity reaction. The increased permeability correlates with the potency of NSAID-induced COX inhibition. Intestinal inflammation caused by NSAIDs can persist for up to 3 years after discontinuation [1].

NSAID-induced colitis often presents with left lower quadrant cramping, followed by bloody diarrhea or rectal bleeding, and urgency to defecate within 24 hours of symptoms onset. Symptoms vary widely, ranging from mild discomfort to severe pain, with or without bleeding, complicating diagnosis. High clinical suspicion is crucial for patients with abdominal pain and/or bloody diarrhea without an infectious cause, as classic features may be absent [5, 6].

The preferred initial test for suspected ischemic colitis is a CT abdomen with IV and oral contrast, recommended by the American College of Gastroenterology within hours of admission. Suggestive findings include bowel thickening, edema, and pneumatosis intestinalis. Other imaging methods like MRI, ultrasound, and x-ray have limited roles and are not routinely recommended [6–8].

Diagnosis should be confirmed with colonoscopy and biopsy whenever feasible. Minimal insufflation and preparation are recommended to reduce the risk of further ischemia or perforation. Typical macroscopic findings include mucosal edema, erythema, erosions, and ulcerations. Severe cases may show deep ulcers or full-thickness colon involvement, potentially leading to gangrene [6, 7].

NSAID-induced mass-forming granulomatous colitis is an exceedingly rare manifestation of NSAIDs toxicity. Our literature search underscores this rarity, as we have identified only one other directly comparable published case of NSAID-induced granulomatous colitis [9]. This report, therefore, makes a notable contribution to the sparse existing literature on this specific adverse event. For comparative analysis, key clinical, endoscopic, and histopathological features of our case alongside the previously reported instance are presented in Table 1.

The presence of a colonic mass, as seen in our patient, was particularly concerning for potential malignancy despite a negative screening colonoscopy 3 years prior [10]. Differentiating NSAID-induced mass-forming granulomatous colitis from a malignant colonic mass is challenging due to overlapping clinical, imaging, and colonoscopic findings. Concerns about malignancy often heighten anxiety for patients and providers. Diagnosis requires tissue biopsy and should be a diagnosis of exclusion after ruling out more serious conditions. We faced a significant diagnostic challenge: the initial biopsy, though technically adequate, showed only inflammatory findings despite strong clinical and radiological evidence suggesting malignancy. This discrepancy prompted a repeat colonoscopy for clarification, highlighting the complexities that arise when histopathology results do not align with a high pretest suspicion for serious conditions.

In nongangrenous NSAID-induced colitis, symptoms typically resolve within 2-3 days [5]. Management focuses on resuscitation, discontinuation of the offending agent, bowel rest, and monitoring for peritoneal signs. Glycemic checks are essential for diabetic patients. Surgical evaluation is reserved for worsening conditions [6, 11]. Nonsurgical approaches include corticosteroids, immunomodulators, or low molecular weight heparin based on comorbidities, though evidence does not favor a specific protocol. Notably, corticosteroids may delay peritoneal sign detection, potentially complicating care [6].

Prompt identification and discontinuation of NSAIDs generally result in a good prognosis. However, the widespread use of NSAIDs and their interactions with other medications in patients with chronic conditions warrant further research into their role in drug-induced colitis. The findings from this case may also have implications for how patients on long-term NSAID therapy are monitored and managed, potentially suggesting a need for increased vigilance or specific surveillance protocols in select cases presenting with unexplained GI symptoms.

## 4. Conclusion

This case emphasizes the importance of considering NSAID-induced complications when diagnosing colonic masses. The presented case of a 63-year-old woman highlights how NSAIDs can cause granulomatous inflammation mimicking malignancy. Early recognition and discontinuation of ibuprofen led to complete resolution, avoiding unnecessary surgery. Clinicians should maintain a high index of suspicion for NSAID-induced injuries, especially in patients with relevant GI symptoms and a history of NSAID use. Careful medication history and judicious NSAID prescription are crucial to prevent serious GI side effects, and further research is needed to improve prevention and management strategies.

## Figures and Tables

**Table 1:** Key clinical, endoscopic, and histopathological features of our case of NSAID-induced granulomatous colitis compared to a previously reported similar case.

Author	Our case	Baert et al. [9]
Year of publication		1995
Age of the patient	63 years	68 years
Gender	Female	Female
NSAID implicated	Ibuprofen	Diclofenac
Duration of NSAID use	Several weeks to months	2 years
Presenting symptoms	Vomiting and diarrhea for 4 days	Acute onset of bloody diarrhea
Colonoscopic findings	Infiltrative, polypoid, ulcerated mass	Deep ulcerations, erythema, and erosions
Location	Ascending colon	Transverse colon
Biopsy findings	Severe acute and chronic mucosal inflammation	Crypt distortion, cryptitis, hemorrhage, fibrosis, and large, nonforeign body-type granuloma
Clinical course	Complete symptoms resolution within 48 h of NSAID cessation	Complete symptoms resolution within 24 h of NSAID cessation
Follow-up	Repeat colonoscopy 6 days later showing complete endoscopic resolution with residual inflammation on histology	Repeat colonoscopy 17 days later showing complete endoscopic and histologic resolution

## Data Availability

The data that support the findings of this study are available on request from the corresponding author. The data are not publicly available due to privacy or ethical restrictions.
